# Binary Solvent Swap Processing in a Bubble Column in Batch and Continuous Modes

**DOI:** 10.1021/acs.oprd.1c00455

**Published:** 2022-03-16

**Authors:** Phillip Roche, Roderick C. Jones, Brian Glennon, Philip Donnellan

**Affiliations:** School of Chemical & Bioprocess Engineering, University College Dublin, Dublin 4, Ireland

**Keywords:** bubble column, mass transfer, continuous manufacturing, evaporation

## Abstract

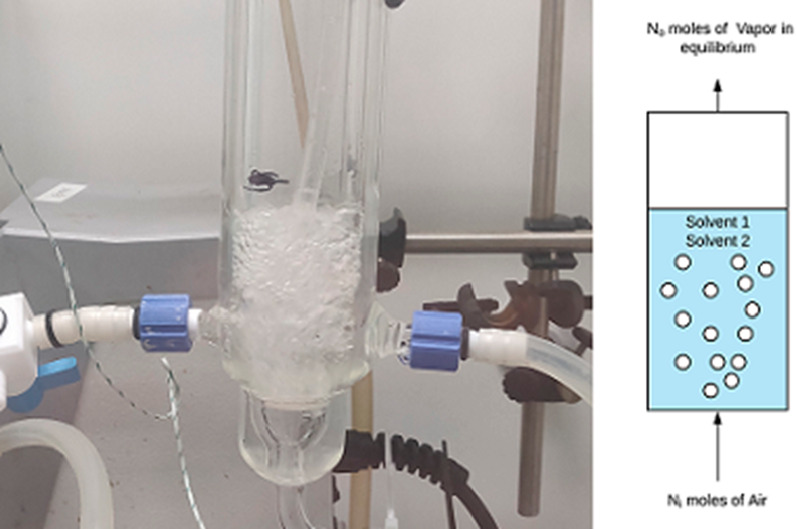

A lab-scale bubble column was investigated as an alternative means to achieve a low-temperature binary solvent swap of solutions containing pharmaceutical materials at atmospheric pressure, for batch and continuous configurations. The rate of solvent evaporation was predicted by first-principles vapor–liquid equilibrium (VLE) thermodynamic modeling and compared to experimentally achieved results. For batch configurations, evaporation rates of up to 5 g/min were achieved at gas flow rates up to 2.5 L/min (0.21 m/s superficial velocity) and temperatures up to 50 °C. This achieved 99 mol % purity of the desired solvent within three “put and take” evaporations from a 50:50 starting mixture. The evaporation rate profiles for the duration of the experiments were calculated, and the changing concentration profile was predicted within satisfactory error margins of <5%. Continuous process modeling explored a multistage equilibrium configuration and could predict the approach to attaining steady-state operation for various operating conditions. All rates of evaporation and resulting changes in solution concentration were measured, and direct comparison of model predictions fell within instrumentation error margins, as previously. This underlined the capability of the model to provide accurate representations of predicted evaporation rates and binary solution concentration changes during operation.

## 1 Introduction

Traditionally, in-demand active pharmaceutical ingredients (APIs) are manufactured at large scale, often in batch vessels on the scale of 4000–8000 L. Large-scale batch manufacturing is attractive because of its reliability, robust processing and cleaning methods, and high yields of product. However, in recent years the pharmaceutical industry has shown much interest in adapting production technologies to continuous modes of manufacturing.^1,2^ Continuous manufacturing is based on developed flow chemistry approaches^3^ and couples numerous unit operations to achieve a controlled throughput of product at all stages of the process. This is achieved by applying various flow control technologies throughout and process analytical technology (PAT) at critical stages of the process to develop an understanding of the performance of the various operations and as a control strategy to ensure good quality and productivity with process feedback loops.^4,5^

In many API manufacturing process chains, required concentration gradients between the various unit operations exist. Often a solvent swap is needed to achieve this desired change in solvent matrix. This solvent swap can be required for key impurity rejection based on crystallization performance, for example, to achieve good purging from the assay or a better enantiomeric excess of the API.^6,7^ According to the current literature, a fully continuous method for evaporative solvent swaps at production scale has yet to be achieved, and various practical evaporation methods are applied in lieu, to avoid bottlenecking and backing up of the otherwise continuous process chain.^8^

The application of a bubble column may be a suitable option to achieve a controlled continuous solvent swap.^9,10^ Aeration and bubbling methods are used extensively in the chemical and biopharmaceutical industry because of the high interfacial areas and good mass transfer that they provide between phases. They are frequently employed in wastewater treatment facilities for the biological oxidation of organic waste and as a method to remove trace volatile organic compounds and ammonia.^11^ Performing as an evaporator, they have also been studied as a potential method for the desalination of seawater at lower temperatures than the boiling point of the saline solutions, yielding a potable condensate.^12^

Bubble columns often follow a conventional design with a sintered frit or sparger at the base where the gas is dispersed into the liquid medium in the form of small bubbles (homogeneous regime^9^). High levels of mixing are achieved by the vigorous introduction of the gas phase into the system. This often results in thermally homogeneous operation between the two phases, minimizing the likelihood of hot-spot formation at heat-exchanging surfaces.^13,14^ The vigorous mixing also achieves good rates of heat and mass transfer between the two phases^15,16^ and often allows the exiting gas stream to reach equilibrium with minimal contact time required between the two phases.^9,10,17^ This saturated vapor is what provides the good rates of evaporation achieved by bubble columns at temperatures appreciably lower than their respective boiling points.

To date there have been no applications of bubble columns to achieve evaporation of process streams in the pharmaceutical or fine chemical production industries. Other techniques to achieve interfacial contact evaporation have been explored, however. Deadman et al.^18^ exposed a high-surface-area spray of a solution to an excess of a solvent-free gas stream in an enclosed heated device. This enabled a solvent swap from pure toluene to a 10:1 v/v ethanol/toluene mixture by mixing of a 0.16 mL/min flow of toluene and product with a 0.64 mL/min flow of toluene upstream and introducing this into the evaporator device. The liquid flow was atomized at the inlet, and an excess 10 L/min flow of nitrogen evaporated the solution in flow. The subsequent 10:1 volumetric ratio of the ethanol/toluene solution was used for the succeeding reaction steps. On a similar scale, Escribà-Gelonch et al.^19^ employed a heated vacuum chamber in a tandem process chain prior to crystallization of vitamin D_3_. The process mixed a solution of vitamin D_3_ in methyl *tert*-butyl ether with acetonitrile, and the vacuum chamber enhanced the rate of mass transfer of liquid droplets as they passed through, improving the yield of the subsequent crystallization. On a larger scale, Johnson et al.^20^ incorporated a 20 L rotary vacuum evaporator into a continuous process operating in semibatch mode with operation parameters analogous to those of a fully continuous process. The unit was capable of high throughputs (5.5 kg/h intermittent flow rate) and achieved a solvent swap of a 1:1.17 ethyl acetate/toluene solution to pure toluene. This was achieved by evaporation to dryness in the rotary chamber and subsequent redissolution in fresh toluene. This was compared to a batch equivalent operation and suggested advantages with regard to process footprint (400 L batch vessel compared with the 20 L unit) and processing time. This ultimately reduced a three-step “put and take” volume method to a single-step solvent replacement. The ability of the evaporator to operate at low temperatures (40–50 °C) was also described as beneficial, as it reduced the likelihood of impurity formation due to unreacted ingressed starting material from the preceding operation.

In the research outlined here, the evaporation rate of a binary solvent system produced by a bubble column under ambient pressure is examined. The effect of the dissolved API on the rate of evaporation is considered and discussed. It has been shown that appreciable rates of evaporation can be achieved by the high rates of interfacial mass transfer between the liquid and gas phases. A thermodynamic model is developed that can predict the rates of evaporation and relative concentration change in the binary mixture within the accuracy of the instrumentation.

## 2 Materials and Methods

### 2.1 Experimental Setup

**Figure 1:**
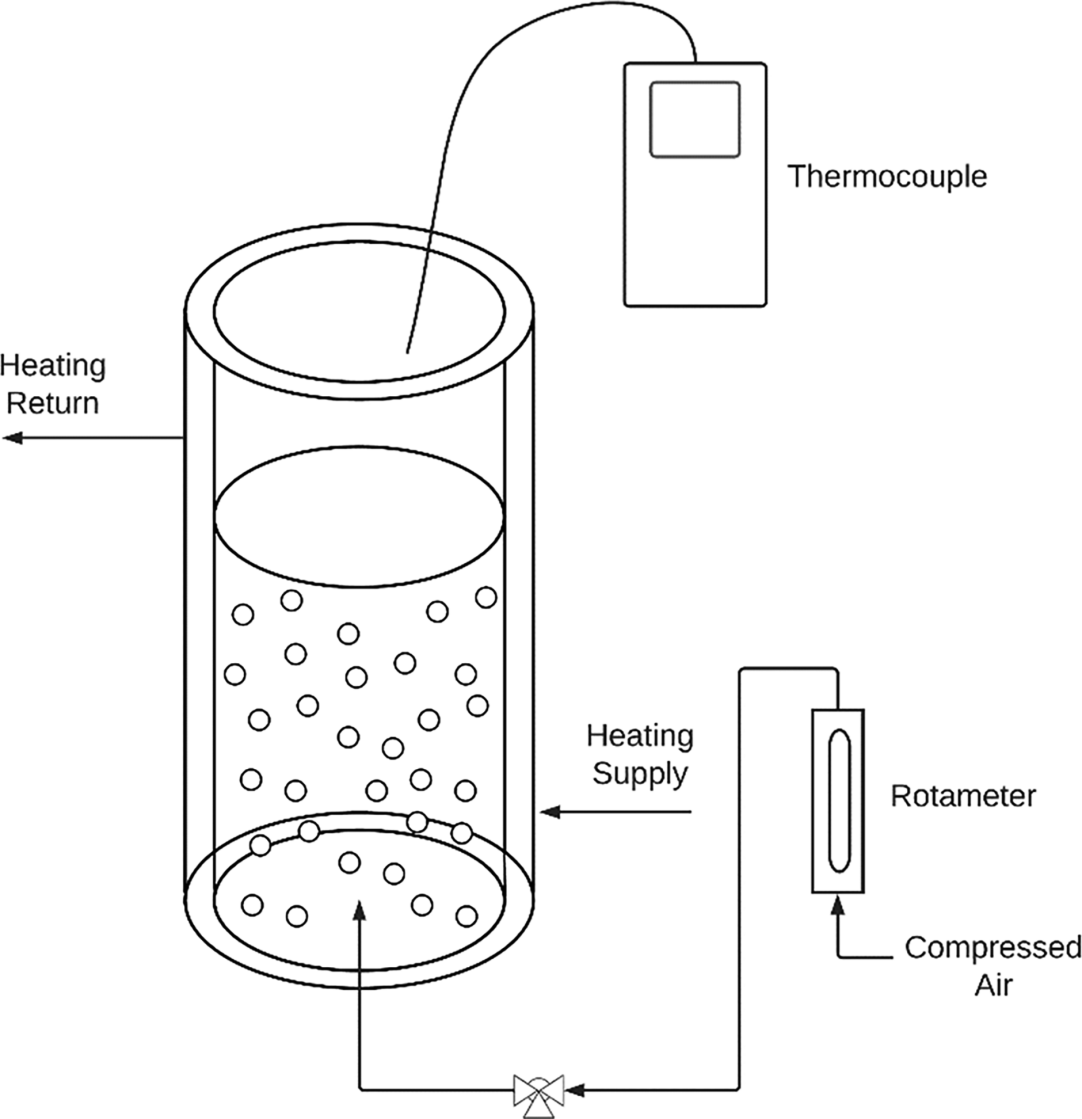
Experimental configuration for solvent swap operations in the bubble column (batch configuration).

The experimental setup for the binary solvent evaporation included a bubble column with a diameter of 0.03 m and a height of 0.4 m (Figure 1). The column was fixed to a retort stand and open to the atmosphere in a fume hood, and it was designed with an annular jacket on the external walls through which a heating medium was passed. This allowed a constant temperature to be maintained for the duration of the experiments using a Julabo FP50-ME circulator set to a desired temperature.

The sintered frit at the base of the column had a porosity grade of 40–100 μm, which dispersed the gas into the liquid as a swarm of fine bubbles. Thermocouples (RS Pro Type-K) measured the liquid temperature and the vapor temperature at the outlet. Air from a dry-air house source regulated at 30 psi was introduced at the base of the column, and its flow rate was controlled with a rotameter (Omega Engineering FL2013, 0.4–5 L/min, ±5% full-scale error). A three-way valve in the gas line allowed the gas to be diverted as desired without the need to adjust the rotameter setting. This ensured that the desired flow rate setting was undisturbed between measurements and experiments.

**Figure 2:**
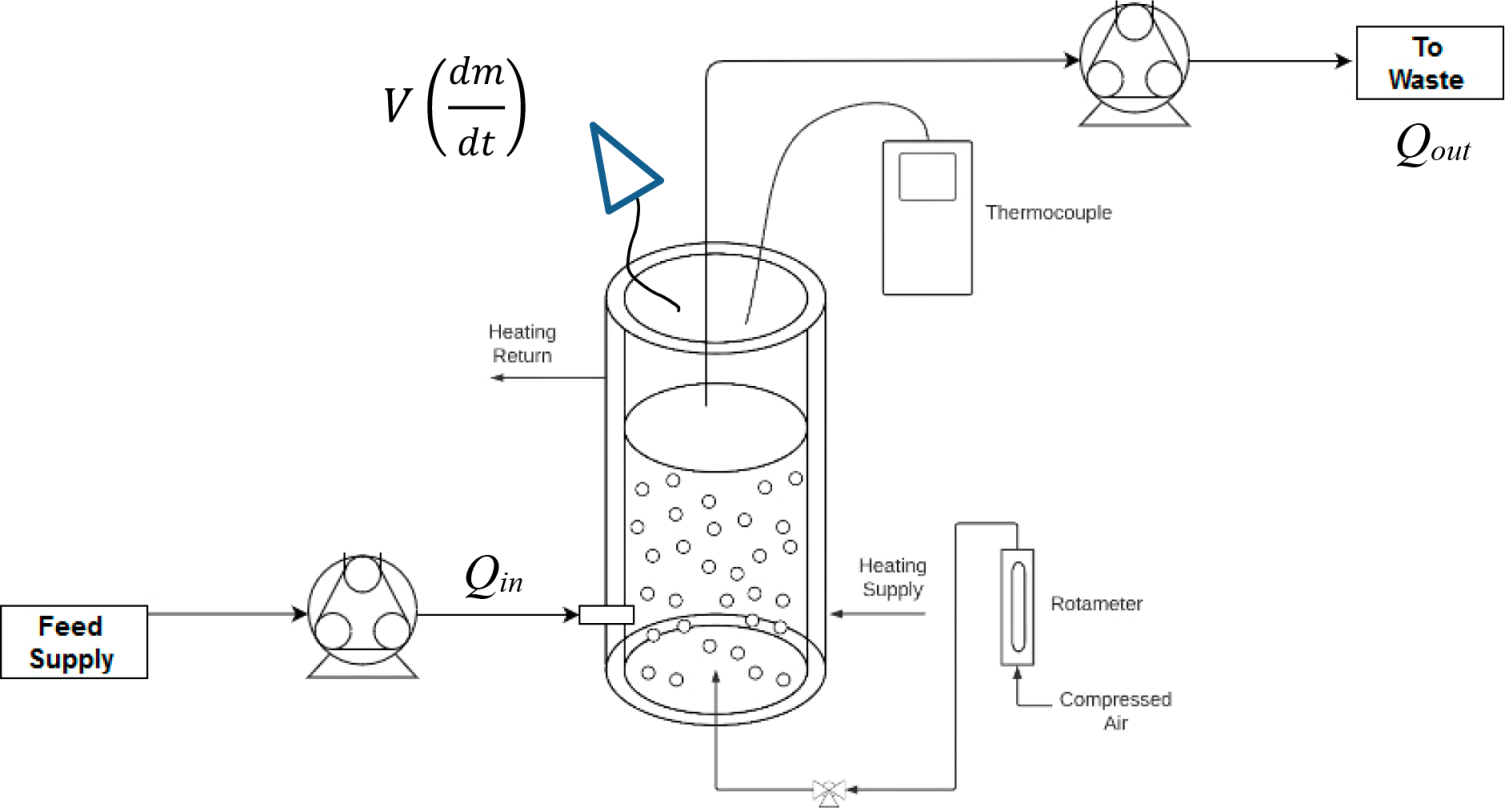
Experimental configuration for continuous solvent swap operations in the bubble column.

The configuration of the batch system was altered to allow for continuous operation, as illustrated in Figure 2. Both pumps were peristaltic, and 1/4″ marprene tubing was used. The feed pump was calibrated to deliver the liquid solution at a known rate. The outlet pump was set to a high RPM value, and the tubing performed as a dip pipe within the column, maintaining constant-volume operation.

### 2.2 Experimental Procedure

At the beginning of each run, the solution weights were measured using an analytical balance (Mettler Toledo XS6002S), and then the liquid was transferred to the column. The desired gas flow rate was set on the rotameter, and the desired temperature set on the Julabo circulator; this was confirmed by a thermocouple within the column and during operation. Initially the gas was diverted by means of the three-way valve as described in section 2.1 to ensure that no losses would occur during the charging to the column. Once the temperature of the solution reached the desired value, the gas was introduced into the column using the three-way valve, and simultaneously a stopwatch timer was started.

At the desired time points, the gas was diverted using the three-way valve, stopping evaporation, and the timer was paused to allow the sample to be taken. A dip tube was inserted into the column, and a peristaltic pump was used to transfer the remaining solution into a Duran flask on a balance, ensuring that no liquid remained in the column following the transfer. The weight of the remaining solution was then recorded, after which the solution was transferred back to the column and the experiment was continued. The samples for concentration analysis were taken by syringe at the desired time points and immediately crimped to minimize losses. Careful attention was given to maintaining the temperature of the solution at the target temperature of each experiment, adjusting the jacket temperature as required.

For the solvent swap batch operation, a known preheated amount of the replacement solvent was prepared to be transferred to the column once the allotted time of the process had passed.

Analytical methods and vapor pressure measurement methods are listed in the Supporting Information.^21,22^

## 3 Mathematical Modeling

### 3.1 Vapor Concentration Modeling

The composition of the vapor of the solvent mixture is known to be a function of the liquid mole fraction of the binary mixture and the temperature. Following the method of Renon and Prausnitz,^23^ the NRTL method was used to predict the activity coefficients for the solvent systems (see the Supporting Information for further details).

### 3.2 Evaporation Model: Batch Operation

**Figure 3:**
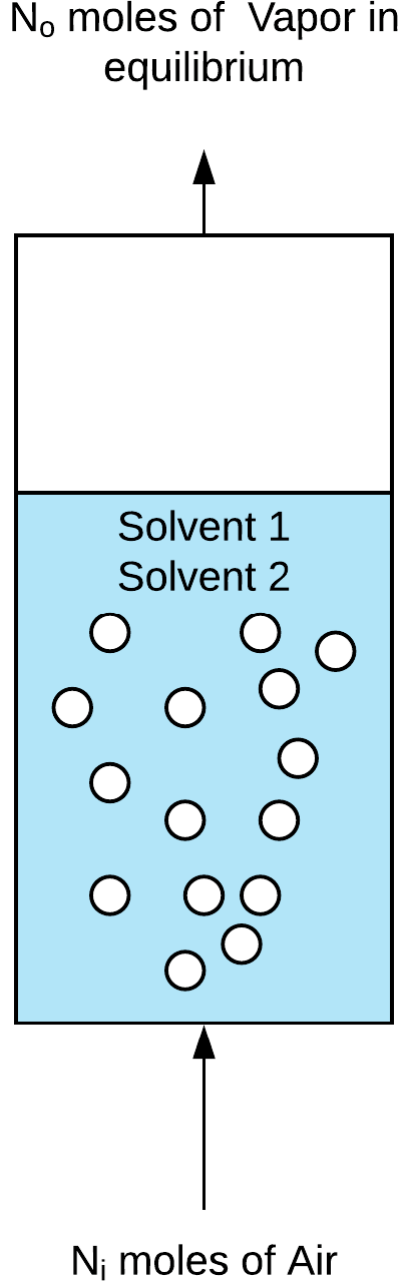
Illustration of the control volume for a mass balance to be applied.

On the basis of the approach developed by Roche et al.,^9^ the evaporation rate of a binary solvent system in a bubble column at a controlled temperature and gas flow rate was predicted with a thermodynamic model from first principles. The approach is generic in that it may be used with any binary solvent system provided that the thermodynamic properties are available, namely, those for vapor pressure predictions and activity coefficient modeling constants. For the batch configuration, a binary solvent solution of known mass and concentration is charged to the column, and the gas flow through the frit in the form of a bubble swarm gives rise to evaporation in the column (Figure 3).

The concentration of solvent 1 in the vapor mixture on a mass basis, *C*_1_ (kg/kg), can be described by the following expression:
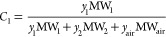
1where *y*_1_, *y*_2_, and *y*_air_ are the vapor mole fractions of solvent 1, solvent 2, and air, respectively (mol/mol), and MW_1_, MW_2_, and MW_air_ are the molecular weights of solvent 1, solvent 2, and air, respectively (kg/mol). The mole fractions of the components in the solution (*x*_*i*_) and the vapor (*y*_*i*_) sum to 1, as shown in eq 2:
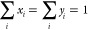
2Therefore, eq 1 can be rewritten as follows:
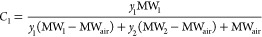
3The vapor mole fraction can be predicted from classical thermodynamics with a known liquid, assuming equilibrium conditions are reached. At equilibrium, it can be assumed that the fugacities of component *i* in the two phases (gas and liquid) are equal:^24^
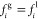
4Applying Raoult’s law to the system and modifying it with the fugacity coefficient to account for the deviation from ideal conditions gives
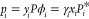
5Where *p*_*i*_ is the partial pressure of component *i* (Pa), *P* is the total system pressure (Pa), ϕ_*i*_ is the fugacity coefficient of component *i*, γ_*i*_ is the activity coefficient of component *i*, and *P*_i_^*^ is the saturated vapor pressure of component *i* (Pa). Under ambient conditions, the fugacity coefficient is equal to 1, and substituting a rearranged eq 5 into eq 3 yields the following expression for the vapor-phase mole fraction:
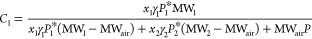
6Equation 6 describes the vapor-phase mass fraction of solvent 1 in equilibrium with the gas phase in the bubble column as a function of the binary-component liquid-phase mole fractions. Similarly, the concentration of solvent 2 on a mass basis can be predicted.

Finally, to calculate the mass-based concentration of the air stream in equilibrium, the mass-based concentrations of solvent 1 and solvent 2 can be subtracted from 1 because only three components make up the vapor system:
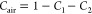
7

Equations 6 and 7 are the basis of the evaporation rate models and will be used in a rate equation to describe the mass transfer of solvent to the gas bubbles over time. It is desired to express the change in mass over time in the bubble column, d*m*/d*t*, and this function can be derived with the assumption of a constant operating temperature and pressure:
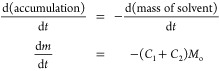
8where *M*_o_ is the mass of vapor leaving the column. As the air is assumed to be noncondensable, the air mass balance throughthe column can be obtained as follows:
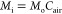
9which can be rearranged to give the rate at which mass leaves the column:
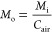
10Inserting eq 10 into eq 8 gives
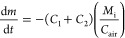
11The first factor in parentheses in eq 11 is the sum of the mass fractions of the two solvents in the gas phase in equilibrium, and the second factor describes the total mass of vapor (solvents and air) leaving the bubble column. Both of these are functions of time. The mass of gas entering the column can be calculated using the ideal gas equation and the known parameters as follows:
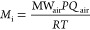
12where *Q*_air_ is the flow rate of air. The evaporation rate was predicted using MATLAB and graphically represented, as can seen in the Results.

### 3.3 Evaporation Model: Continuous Operation

The configuration of the batch system was altered to allow for continuous operation, as illustrated in Figure 2. The model developed in section 3.2 applies in this configuration also, but the feed rate and outlet flow rate are also taken into account in a component mass balance as shown:
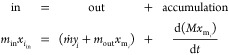
13where *m*_in_ and *m*_out_ are the mass flow rates of the solvent solution in and out, respectively (kg/s), *ṁ* is the predicted total rate of vaporization leaving the system (kg/s), *M* is the instantaneous amount of mass in the system (kg), *x*_*i*_in__ and *x*_m_*i*__ are the instantaneous mass concentrations of solvent *i* in the feed and in the system, respectively, and *y*_*i*_ is the mass concentration of *i* in the vapor. The operating volume may be assumed to be constant in continuous mode, as shown by the experimental configuration in Figure 3, because the dip pipe is in a fixed position. Knowing the solution density profile across the range of operation concentrations allows a simple conversion between mass and volume as required. Applying the condition of constant mass to eq 13 by assuming that the density change across the integral step does not impact the system mass significantly allows the mass to be isolated from the differential, as shown in eq 14:
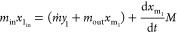
14Next, eq 14 is rearranged and integrated across the time iterative step for the change in concentration in the liquid:
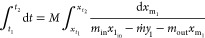
15Setting *t*_1_ = 0 and substituting the outlet flow rate (*m*_out_) by the difference between the inlet flow rate and the evaporation rate (*m*_in_ – *ṁ*) gives eq 16:
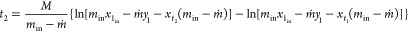
16The evaporation rate of solvent 1 (*ṁy*_1_) is a function of the instantaneous concentration of liquid and can be expressed as such using eq 6 if desired. Here it is left in terms of the vapor mass fraction *y*_1_ for clarity. Simplification gives
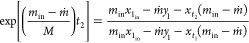
17Rearranging eq 17 gives the following expression for *x*_*t*_2__, the concentration of the liquid for the next time point:
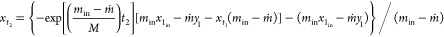
18Equation 18 allows liquid concentration changes to be predicted across the time increment, which is to be based on the unit of flow rate of gas to the system (i.e., per second). This new predicted concentration value can then be taken as the base value for the next time point for calculating the equilibrium conditions, in particular the associated change in vapor concentration and selective evaporation rates. By the use of the expressions outlined in this section, a time-iterative loop can be run using MATLAB to predict the approach to steady-state conditions.

## 4 Results and Discussion

### 4.1 Batch Swap Results

#### 4.1.1 Ideal System: Acetone/IPA

**Figure 4:**
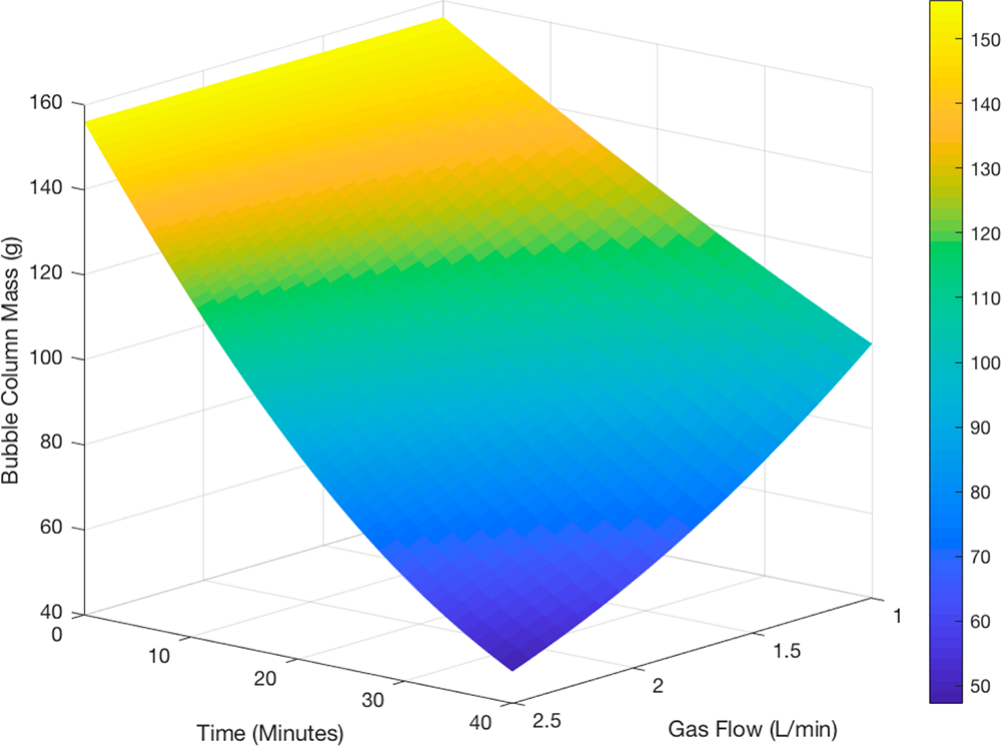
Surface plot showing the range of achievable evaporation rates of acetone and IPA solution as a function of time and gas flow rate, modeled at a fixed temperature of 40 °C. An initial mass of 158 g is representative of 200 mL of a 1:1 mol/mol acetone/IPA mixture. The experiment was modeled over a 40 min time duration (superficial velocity range: 0.085–0.21 m/s).

**Figure 5:**
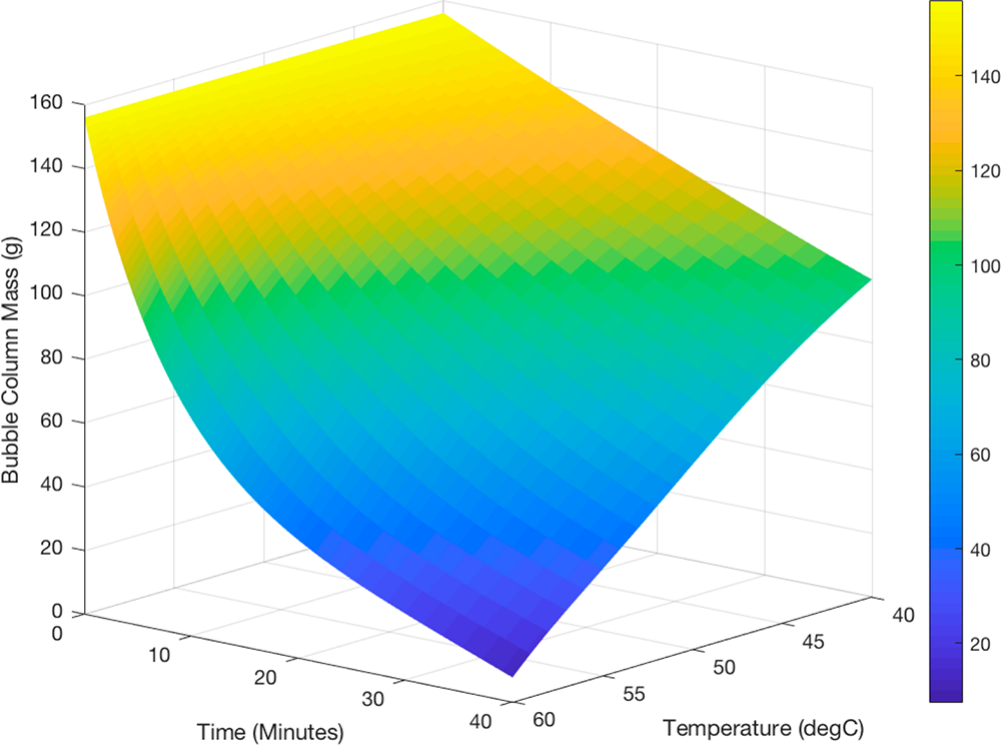
Surface plot showing the achievable evaporation rate of acetone and IPA as a function of time and temperature from the derived model at a consistent gas flow rate of 1 L/min (0.085 m/s). The initial mass is the same as in Figure 4.

An ideal binary solvent system, in this case one that does not form an azeotrope, was studied in batch configuration, as described in section 3.2. Acetone and IPA were chosen for this study because no azeotrope is formed between them, they have relatively low toxicities, and they are common solvents to the pharmaceutical industry. Figures 4 and 5 show the modeled rates of evaporation of acetone and IPA mixtures over the ranges of gas flow rate and temperature that were experimentally tested.

**Figure 6:**
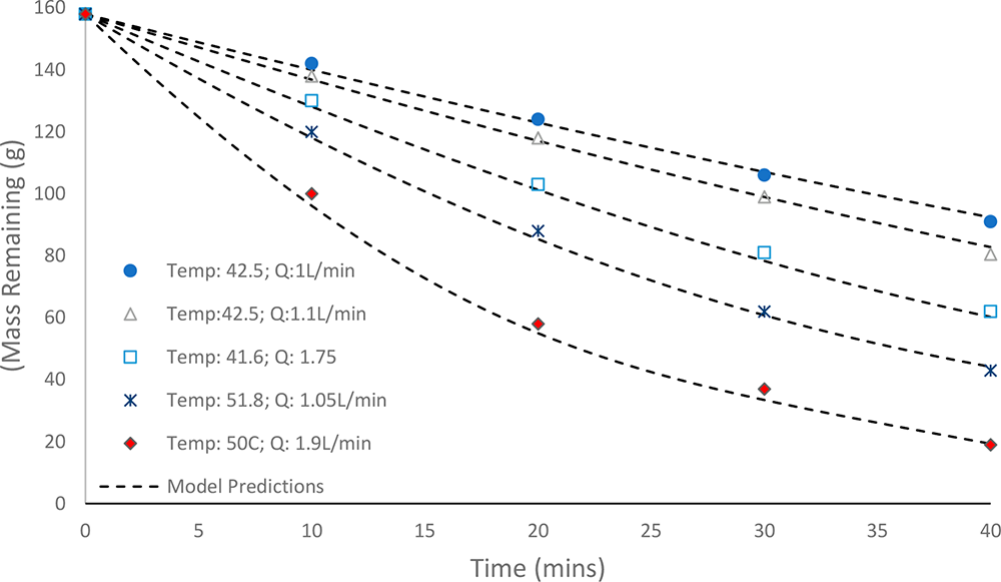
Comparison of model predictions of evaporation rate and experimental data points investigated under known constant temperatures and gas flow rates. The black dashed curves show the model predictions for the various operating parameters corresponding to the different experimental runs (superficial velocity range: 0.085–0.16 m/s).

**Figure 7:**
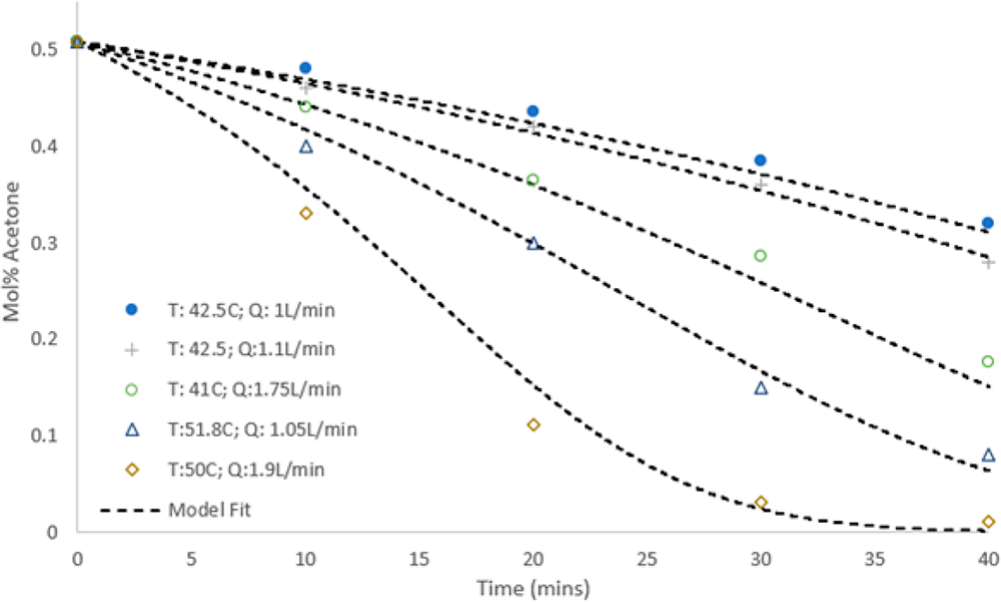
Comparison of the model predictions and experimental data points for the liquid-phase mole fraction of acetone. The black dashed curves show the model predictions for the various operating parameters corresponding to the different experimental runs.

The more volatile component, acetone, was evaporated preferentially over time, and the effect is seen as the rate of evaporation decreases as the solution becomes enriched in the less volatile component, IPA. To experimentally validate the developed models, batch experiments were performed from the same starting conditions. The results from numerous batch evaporation experiments are shown in Figures 6 and 7, with the results of the predictive model from section 3.2 overlaid. The model closely follows the data for the different test conditions over the entire experimental space in both mass and mole fraction. The normalized root-mean-square deviation (NRMSD) values for the sets of experiments in Figures 6 and 7 were calculated as 3.97% and 2.6%, respectively. The NRMSD was chosen as the most appropriate comparison between modeling and experimental measurements because of the evidently nonlinear model profiles. The errors were found to be satisfactorily within the instrumentation error of <5%, as expected from the gas rotameter. The goodness of the fit of the model to the experimental data suggests that the gas leaves the column saturated and in equilibrium with the predicted changes in liquid mole fraction. This highlights that the assumption of minimal resistance to mass transfer on the gas side is valid under the tested conditions.^9,10^ The model can therefore be applied as a generic approach for predicting the rate of evaporation with known vapor–liquid equilibrium data and fixed process parameters.

**Figure 8:**
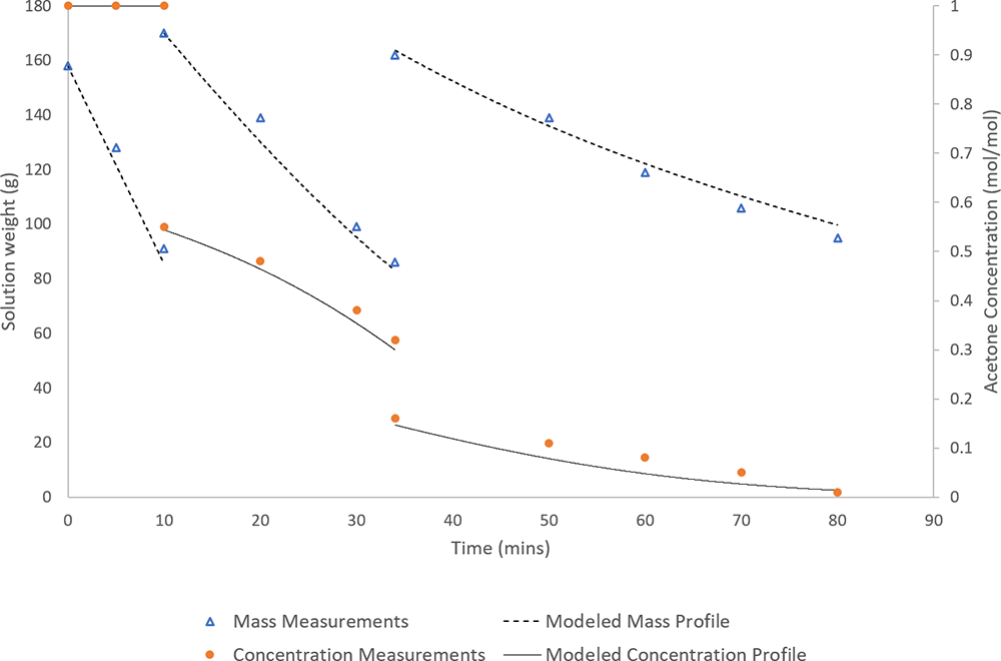
Batch swap of acetone to IPA at *T* = 40 °C and a gas flow rate of 2.5 L/min (0.21 m/s). The initial step starts with pure acetone, which is replaced by IPA for the second step. By the end of the third step, 99% purity is achieved in the reduced equivalent, and this can be brought to further purity by replacement of the removed volume with IPA.

A swap between acetone and IPA was carried out in batch mode following the previous results and to test the robustness of the modeling approach across the full concentration range. The swap was carried out stepwise from pure acetone, and a put-and-take method was used to swap to IPA. When a desired amount was evaporated off, fresh IPA was charged to make the column contents back up to the original volume. This procedure was repeated for two volume replacements, and the results are shown in Figure 8.

As shown in Figure 8, the first evaporation stage is described by a distinct linear profile. This is known to be the case for pure-solvent evaporation systems.^9^ The acetone was steadily removed for 10 min, and the concentration remained unchanged because it was a pure liquid. Then 79 g of IPA was charged to the system, and the concentration fell to a value of 55% mol/mol (55.8% w/w), as measured by GC. Following the charge of IPA, the evaporation process resumed, and the equations from section 3.2 describing the binary solution batch evaporation process were applied. The results of the predictive model are overlaid on the experimental data in Figure 8 for the overall experiment. This was repeated a third time, and the desired purity of <1 mol % acetone was achieved by the end of this stage. The effect of the decrease in volatility of the solution over the duration of the experiment is evident by the decrease in the evaporation rate as the acetone is removed, enriching the solution in IPA.

#### 4.1.2 API Solution: Acetone/IPA Swap

**Figure 9:**
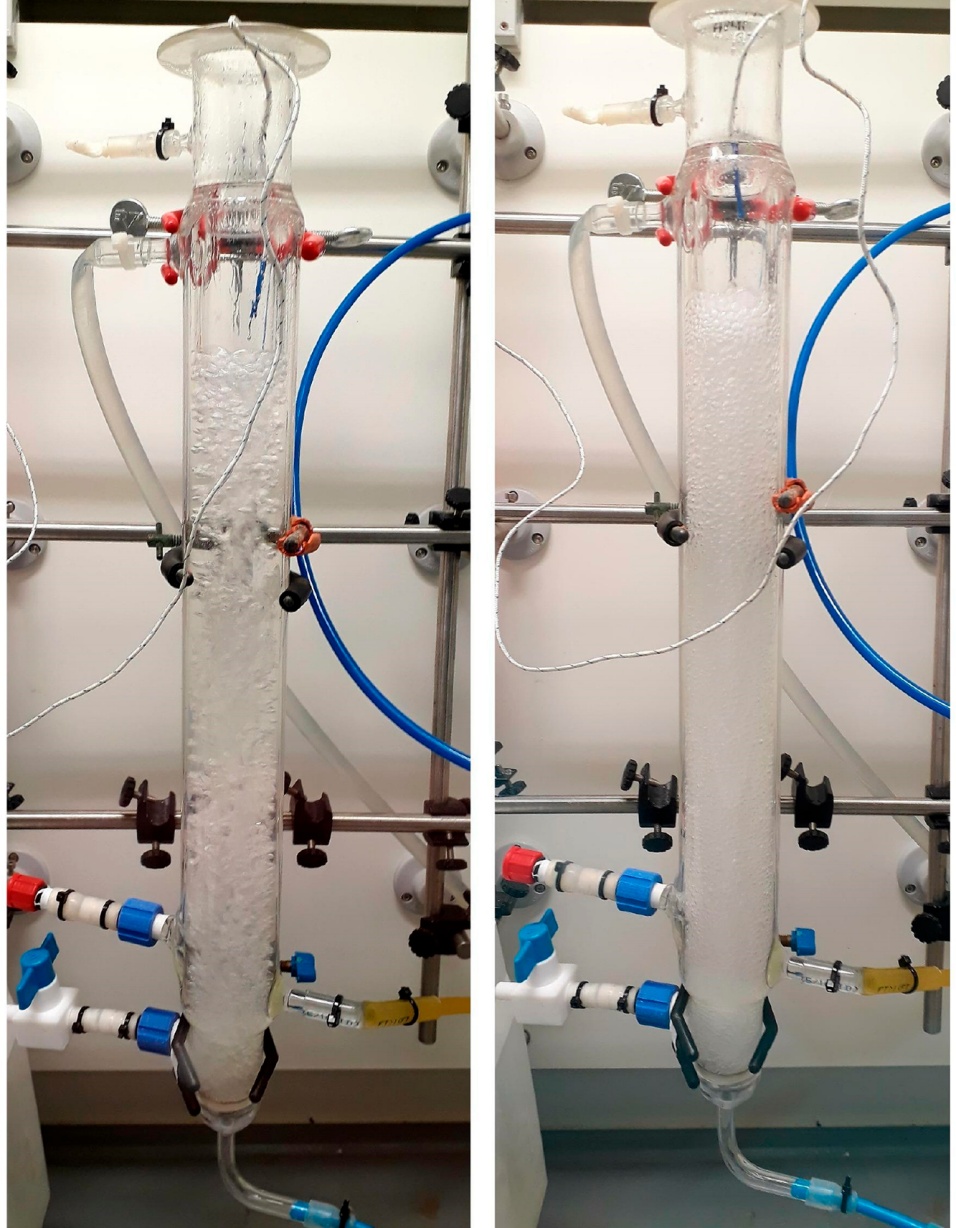
Images of the bubble column operating with (left) pure solvents and (right) dissolved API, operated under same conditions and flow rates.

A nonvolatile solid dissolved in a liquid solution will influence the physical properties of the solution. The nonvolatile molecules located at the gas–liquid interface impose a diminishing effect on the evaporation rate, and the vapor pressure is reduced.^25^ The presence of the dissolved solute also gives rise to surface tension gradients throughout the system, which can cause the solution to foam when sparged with gas, as shown in Figure 9. The foaming, while visibly evident, was not excessive, and the experiment was carried out successfully under the desired conditions.

**Figure 10:**
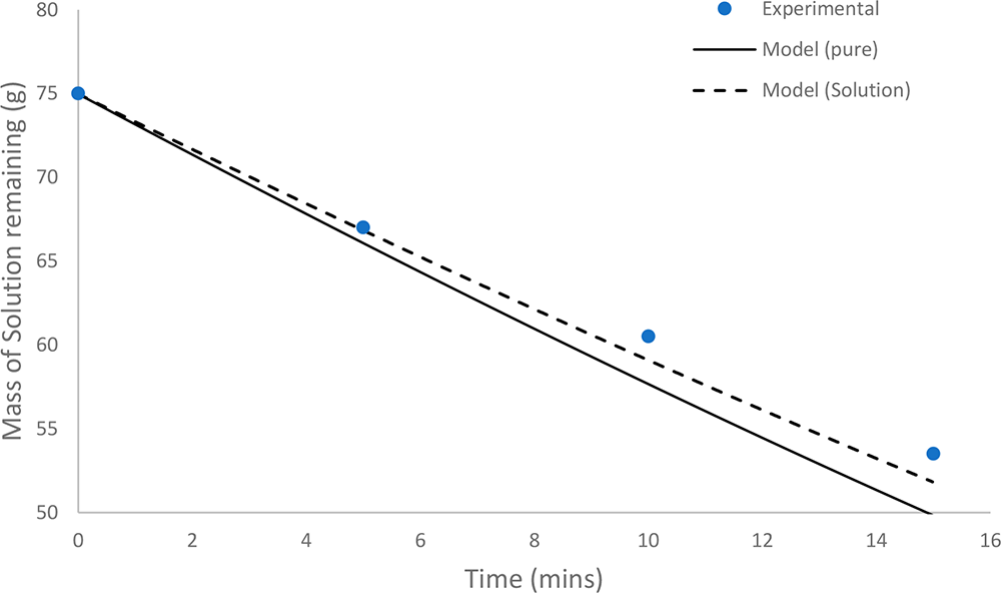
Comparison of experimental results to model predictions for batch evaporation of a 5 wt % solution of paracetamol in 1:1 w/w acetone/IPA. *Q* = 1.15 L/min (0.097 m/s); *T* = 39.3 °C.

**Figure 11:**
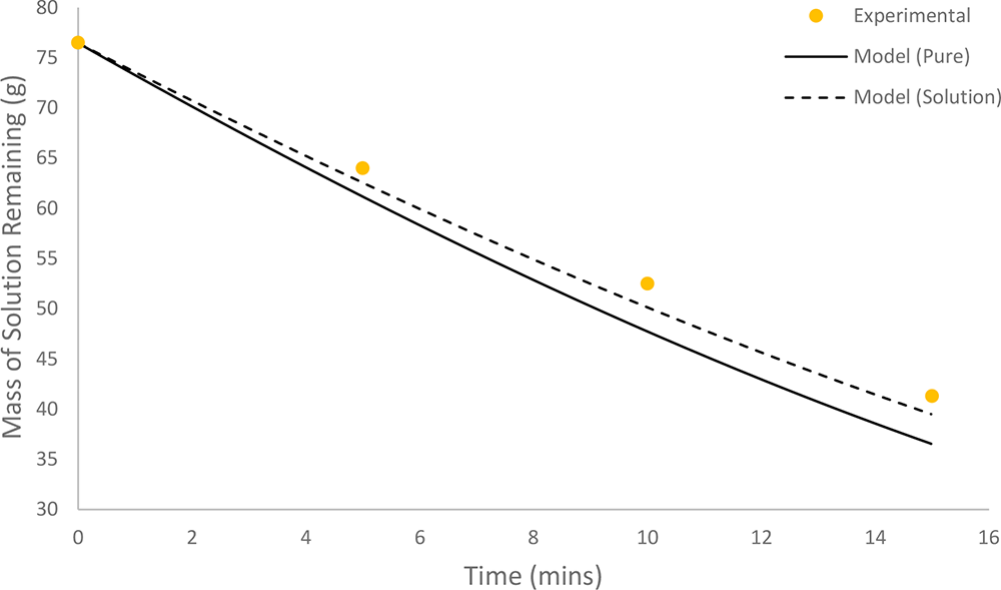
Comparison of experimental results to model predictions for batch evaporation of a 5 wt % solution of paracetamol in 1:1 w/w acetone/IPA. *Q* = 1 L/min (0.085 m/s); *T* = 48.1 °C.

From previous work,^10^ it was known that the introduction of a dissolved solute would cause a reduction in the vapor pressure of the solvents, and this effect would need to be measured. Comparing the pure vapor pressure profile to that of the reduced vapor pressure, a reduction in vapor pressure was measured as appreciably constant across the entire range of temperatures tested for both acetone and IPA at a dissolved solute concentration of 10%. The Supporting Information gives further details on the measurement of the reduced vapor pressure using an isoteniscope. Results from the batch experiments are shown in Figures 10 and 11, where the measured data are shown as discrete points and the predictions of the two models are shown as continuous and dashed lines.

As the solution becomes more concentrated in the dissolved API, the effect of the reduction in the evaporation rate becomes more pronounced. The modified model accounting for the reduction in vapor pressure increases the accuracy of the evaporation rate prediction. By direct comparison of the two profiles, the improvement in the accuracy of the predicted rate of evaporation is evident, as the reduced model approaches the experimentally measured points more closely compared with the pure system model. For the experiment described by the profile in Figure 10, the reduced model yielded an NRMSD of 4.73%, compared with 9.38% for the pure system model, corresponding to a reduction in the error margin by 4.65%. For the experiment shown in Figure 11, the reduced model yielded an NRMSD of 4.47% compared with 9.15% for the pure system model, resulting in a reduction in error margin by 4.69%.

**Figure 12:**
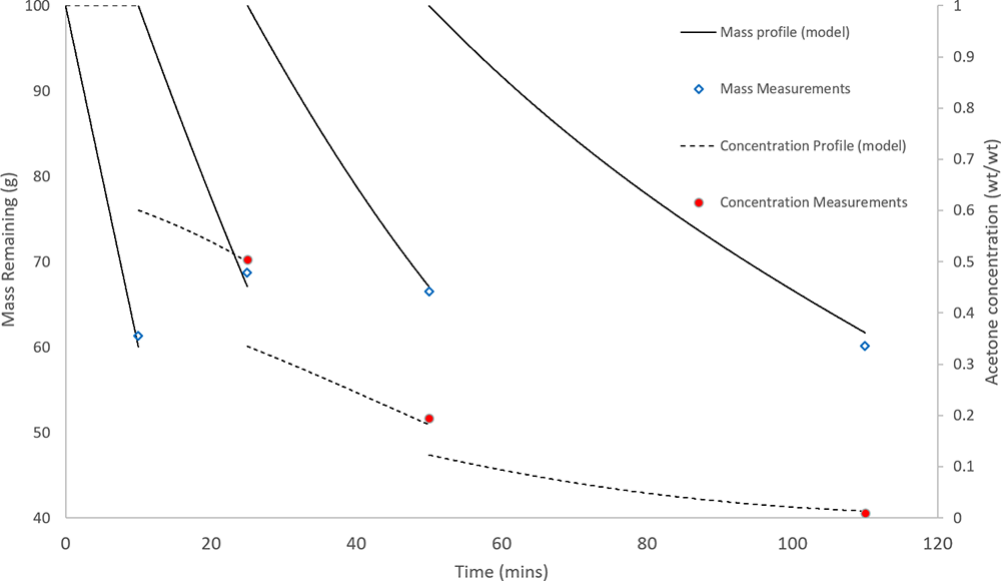
Put-and-take solvent swap of a 5 wt % solution of paracetamol in acetone to IPA. Process durations were predicted with the developed models, and the process was paused at the desired time points for sampling and replacement of the desired solvent. All modeling here uses the reduced vapour pressure measurements.

Building on the single-solvent batch experiments, a solvent swap experiment was performed. It was intended to swap a 5 wt % solution of paracetamol in acetone to IPA by the put-and-take method in a single column, as described in section 4.1.1. The batch evaporation model was applied here for all model predictions, and the reduced vapor pressure estimation method was also applied. The process was run with a 1 L/min flow of gas at a constant temperature of 45 °C. The results of the experiment are shown in Figure 12. When the desired time point had been reached (as estimated by model prediction), the gas supply was diverted via the three-way valve, stopping evaporation. The column was then drained, and its contents were weighed and sampled for concentration measurement by GC. To maintain the solution within the solubility limits of paracetamol in acetone/IPA, the system was constrained to a maximum concentration of 10 wt % for the experimental duration.^26,27^ The evaporated mass measured was replaced by the desired solvent, IPA, back to the original starting mass of 100 g.

Overall, the model provided a good representation of the mass and concentration profiles of the experiment. Error margins of 3.28% and 4.54% were achieved for the mass and concentration measurements, respectively, by NRMSD analysis. The experiment achieved a solvent swap to IPA with >99% purity, with three volume replacements by put-and-take evaporation of the solution, confined to the solubility limits of maintaining a maximum dissolved solids concentration of 10 wt %.

### 4.2 Nonideal System: Batch to Continuous Operation

#### 4.2.1 Batch Experiments

**Figure 13:**
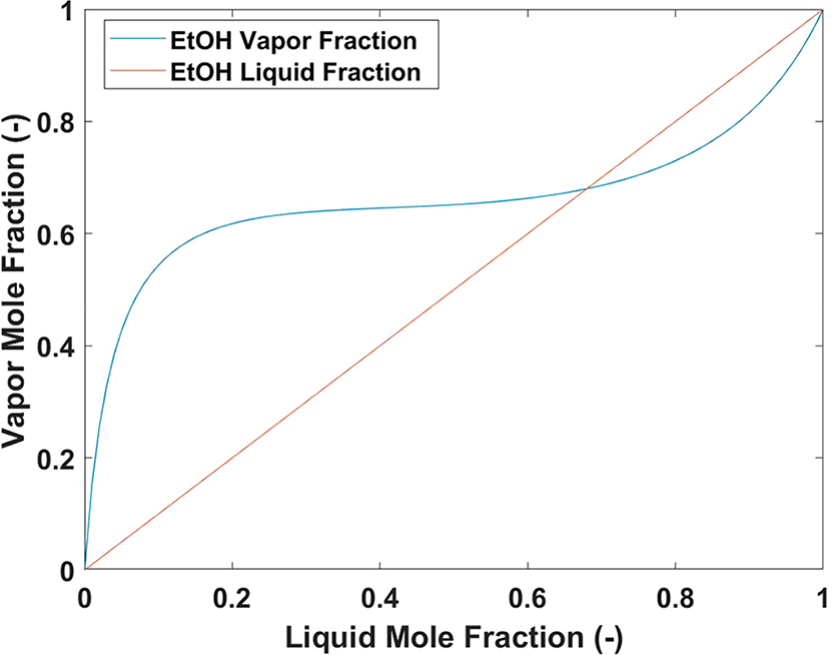
Vapor–liquid equilibrium mole fraction diagram for ethanol and toluene at 25 °C and 1 atm. Activity coefficients were predicted using the NRTL model.

A system that created an azeotrope, ethanol/toluene, was studied here. By the use of the NRTL equation, the *y* versus *x* diagram could be created for the desired temperature across the full concentration range, as shown in Figure 13. According to these vapor–liquid equilibrium results, the azeotrope exists at *x*_EtOH_ = 0.68 for a system at atmospheric pressure at 25 °C. For a batch system operating at concentrations below this, the system will become enriched in the less-volatile component (LVC), toluene. However, above this concentration the system will become enriched in the more-volatile component (MVC), ethanol. This makes a solvent swap from toluene to ethanol (i.e., from a higher-boiling-point solvent to a lower-boiling-point solvent) achievable, which would be impossible if the solvent system were zeotropic.

**Figure 14:**
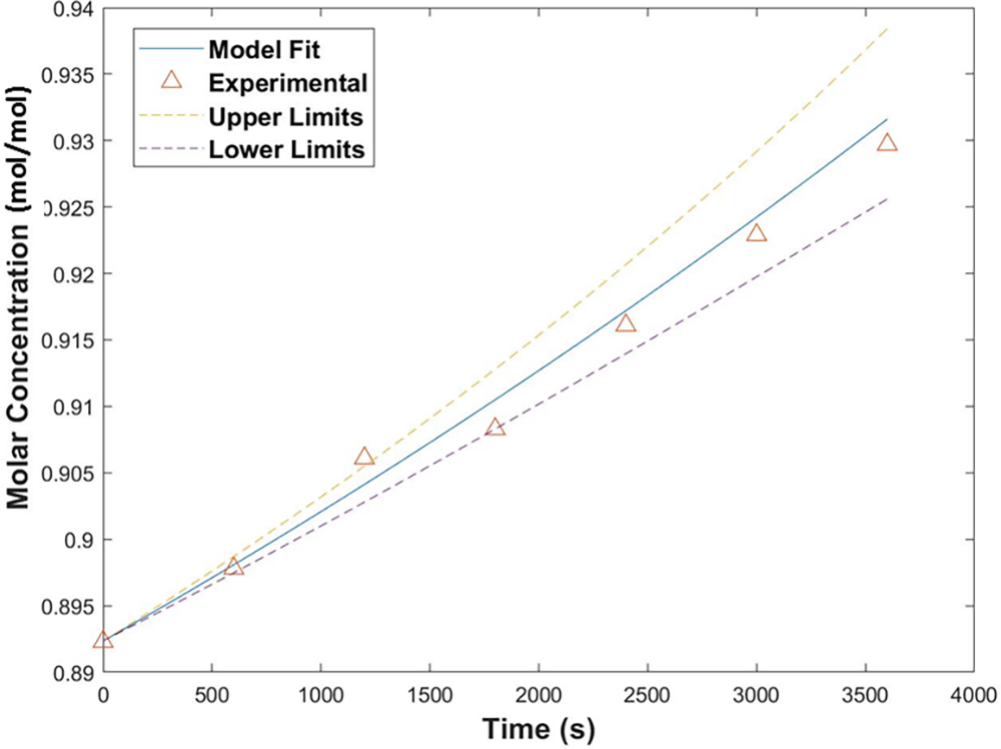
Increase in the concentration of EtOH over time during the batch experiment on the enrichment side of the azeotrope. The dashed lines represent the upper and lower errors for model prediction.

A batch experiment was carried out on a 1:4 w/w toluene/EtOH mixture, which is enriched in ethanol by evaporation. This initial mixture lies on the ethanol enrichment side of the azeotrope and is expected to become concentrated in the MVC. A 100 mL solution was prepared from 64 g of EtOH and 15.96 g toluene, and this was transferred to the column. The experiment was run at a gas flow rate of 2.5 L/min (0.21 m/s) at 25 °C. Samples were taken at known time points and analyzed offline on the GC to determine their concentrations. The results are shown in Figure 14. The model was able to predict the increase in EtOH on the enrichment side of the azeotrope, and concentration of the MVC was achieved. Included in Figure 14 are the upper and lower error margins suggested by the instrumentation error on the flow meter. Evaporation rates of over 0.5 g/min at a gas flow rate of 2.5 L/min (0.21 m/s) were achieved. This rate of evaporation is particularly attractive, as it once again suggests that the thermodynamic maximum rate is reached by a saturated vapor stream exiting from the system.

**Figure 15:**
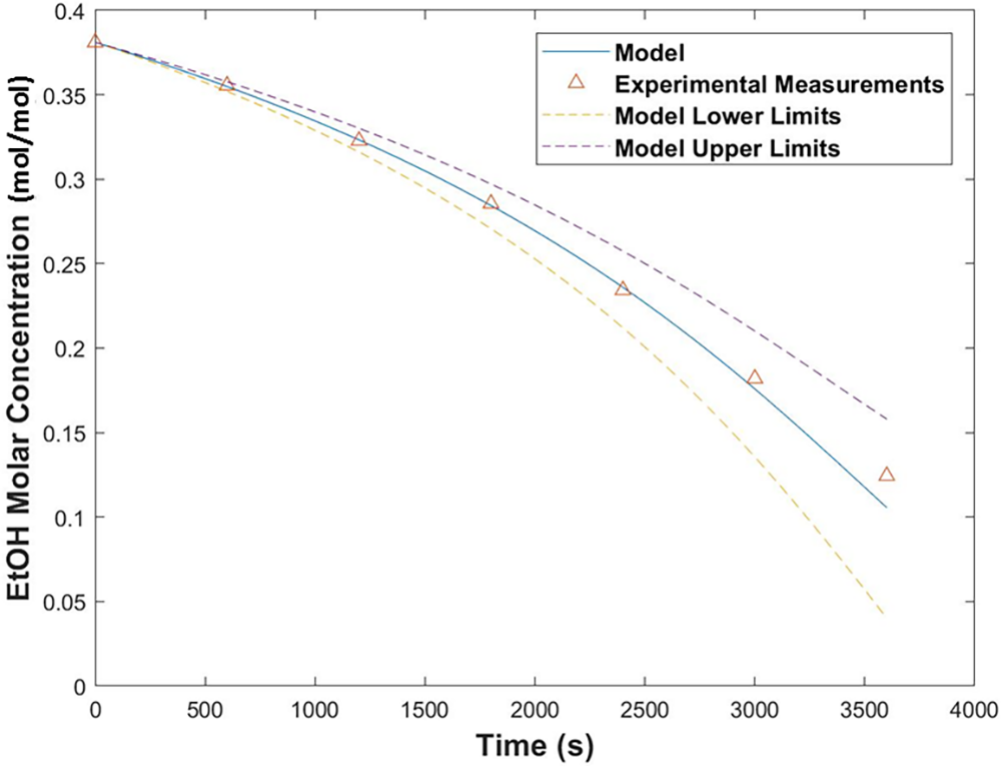
Decrease in concentration of EtOH over time during the batch experiment on the non-enrichment side of the azeotrope. The experiment was performed at 25 °C and 2.5 L/min (0.21 m/s).

The experiment was repeated with an initial solution concentration on the other side of the azeotrope to verify the enrichment phenomenon of the LVC. A 100 mL solution was prepared from 20 g of EtOH and 65 g of toluene (38 mol % EtOH), and the experiment was performed under the same conditions as the previous one. The results are shown in Figure 15.

The results indicate that it is impossible to achieve full enrichment in EtOH or toluene under ambient conditions in the batch configuration from initial conditions on the unfavorable sides of the azeotrope. However, it is possible to operate with continuous processing to achieve enrichment in either solvent by exploiting steady-state operation conditions and multistage configurations, as discussed in the next section.

#### 4.2.2 Continuous Operation

**Figure 16:**
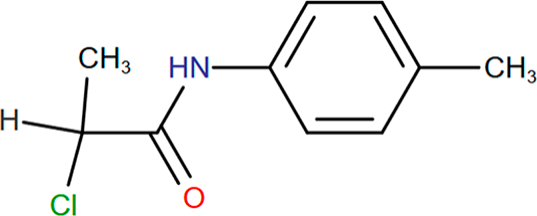
Structure of 2-chloro-*N*-(4-methylphenyl)propanamide (CNMP).

This work was based on a case study performed by this group^28−30^ in which 2-chloro-*N*-(4-methylphenyl)propanamide (CNMP) (Figure 16), a key intermediate for an α-thio-β-chloroacrylamide, which belongs to a class of compounds that have gained a lot of interest in the literature as synthetically versatile APIs.^31,32^ This was produced continuously though a biphasic reaction system; the product was retained in the organic phase (toluene), while the aqueous phase was continuously removed at a controlled rate. CNMP was crystallized from the organic phase continuously to yield the solids for filtration and isolation before redissolution in EtOH for the next step in the reaction process chain. It was proposed that the bubble column could swap between the two solvents continuously, circumventing the necessity for crystallization and filtration and thereby directly improving the yield, as no product would be lost to unrecoverable mother liquor waste streams.

The flow rate of the organic phase, a 3.7 wt % solution of CNMP in toluene, was 7.5 mL/min. This was modeled as a multistage equilibrium system in which the toluene stream was contacted with a stream of EtOH to bring the system to the enrichment side of the azeotrope, allowing it to be concentrated in the MVC. The solution was initially studied in the isoteniscope to account for the vapor pressure reduction, as the concentration of CNMP was to be maintained constant for every stage. At this concentration of CNMP, no measurable vapor pressure reduction effects could be seen, and the system was modeled as such. Also, as it was understood that CNMP is appreciably more soluble in EtOH than in toluene, no precipitation of solid was expected to occur at any stage of the process.

**Figure 17:**
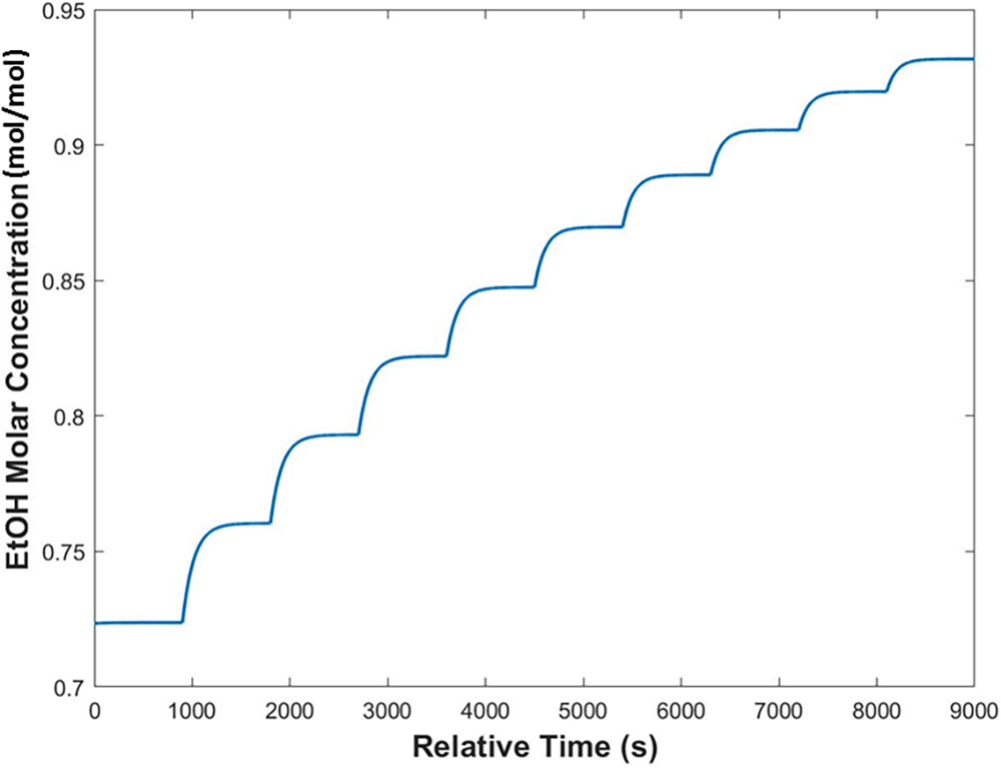
Increase in the concentration of EtOH as a continuous process, modeled in MATLAB over a 10-step equilibrium stage.

A 10-step multistage system was modeled, as shown in Figure 17. This system was modeled with an initial flow rate of 15 g/min (6.5 g/min toluene and 8.5 g/min EtOH), a gas flow rate of 2.5 L/min, and a temperature of 40 °C. The concentration of dissolved solute was maintained constant throughout by feeding to each stage an amount of EtOH equivalent to that removed by the predicted evaporation rates.

**Figure 18:**
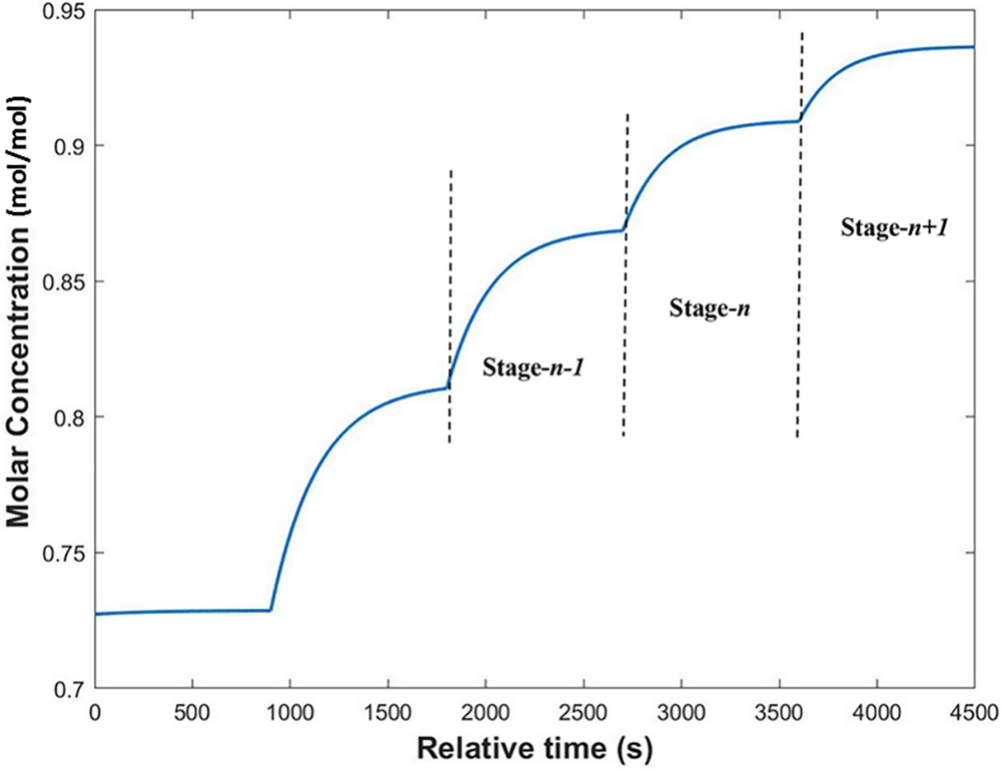
Increase in the concentration of EtOH over time with reduced throughput. The number of equilibrium stages required is reduced to five.

The system achieves an increase in the EtOH mole fraction from 0.725 to 0.925 over a 10-stage equilibrium system for a solution throughput of 15 g/min. The number of stages required was unrealistic for a full swap to be achieved on a lab scale. However, a benefit of continuous manufacturing is the ability to match the productivity of large-scale manufacturing simply by running for a longer duration at lower throughputs. The flow of the feed stream was reduced to 7 g/min (3 g/min toluene and 4 g/min EtOH) and run under the same conditions of gas flow rate and temperature. The results are shown in Figure 18.

**Figure 19:**
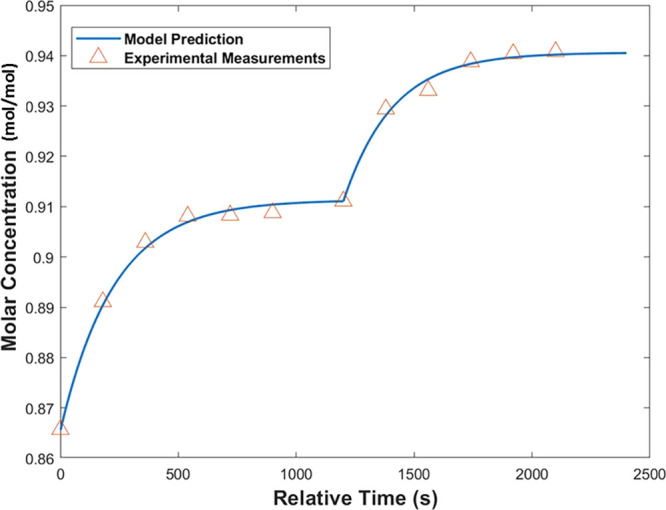
Modeling of stages *n* and *n* + 1 with experimental data overlaid. Experimental samples were taken every 3 min.

An approach to verifying this model experimentally was carried out under the assumption that if the model could predict the performance of two stages in experimental tandem, it would be a reliable representation of the entire system. The final two stages shown in Figure 18 (*n* and *n* + 1) were experimentally performed with a system shown schematically in Figure 2 and run under the same conditions of flow rate and temperature as previously modeled. The feed to stage *n* was prepared on the basis of what was predicted for the outlet of stage *n* – 1: a feed flow rate of 7.5 g/min at an EtOH mole fraction of 0.865. The outlet of this stage was fed to a second column, stage *n* + 1, operating under the same conditions, with a makeup of pure EtOH at 1.65 g/min to replace the evaporated solvent. The results are shown in Figure 19. It was noted that although the CNMP concentration was diluted by the addition of EtOH at each step, it would be feasible to include a controllable concentration step at the end of the chain to achieve the desired concentration for the succeeding reaction.^9^ No precipitation of CNMP was observed throughout the process, which ran for 2 h unimpeded. The EtOH mole fractions from stages *n* and *n* + 1 were measured at the end of the experiment and found to be 0.9089 and 0.9455, respectively. This was a satisfactory result, as it showed that the process was capable of reliably achieving equilibrium conditions over a long duration of operation. The system achieved a throughput of 13.32 g of CNMP in 2 h, which was verified gravimetrically from accumulation of the stage *n* + 1 outlet stream.

## 5 Conclusions

A thermodynamic model was developed to describe the rate of evaporation of a binary solvent mixture in a bubble column and verified experimentally to be accurate under all conditions studied. The system can accurately describe the preferential rate of removal of relatively volatile components based on thermodynamic predictions of vapor pressure using Antoine parameters and NRTL activity coefficient modeling. A solvent swap between two ideal solvents (acetone and IPA) from a low boiling point to a higher boiling point in a batch configuration was achieved. A dissolved API also brought about a reduction in predicted vapor pressures, which was experimentally measured and taken into account in the model, giving rise to higher accuracies. A nonideal system that forms an azeotrope (EtOH/toluene) was also studied. It was found that the negative azeotrope gave an opportunity to enrich the solution in the MVC, ethanol in this case. This was exploited in a multistage continuous configuration, and a solvent swap from a toluene solution of CNMP to >95% enrichment in EtOH was achieved. This configuration had a lower capacity of throughput compared with the alternative method of achieving a solvent swap (i.e., crystallization, isolation, and redissolution), but it reduced losses of product yield that are often encountered in crystallization processes.

## Supporting Information Available

Mathematical VLE modeling, vapor pressure measurement, and analytical measurement methods (PDF)

## Nomenclature

API: active pharmaceutical ingredient
CNMP: 2-chloro-*N*-(4-methylphenyl)propanamide
EtOH: ethanol
GC: gas chromatography
IPA: isopropyl alcohol
LVC: lesser volatile component
MVC: more volatile component
NRMSD: normalized root-mean-square deviation
NRTL: non-random two liquid
PAT: process analytical technology
RPM: rotations per minute
VLE: vapor–liquid equilibrium
