# Supplementary material for: Binary Solvent Swap Processing in a Bubble Column in Batch and Continuous Modes
Source: Org Process Res Dev. 2022 Mar 16;26(4):1191–201. doi: 10.1021/acs.oprd.1c00455 (PMC9016759; doi:10.1021/acs.oprd.1c00455)
Supplement: Supplementary file 1 — op1c00455_si_001.pdf [file op1c00455_si_001.pdf]

Supporting Information

Binary Solvent Swap Processing in a Bubble Column in Batch & Continuous Modes

*Phillip Roche, Roderick C. Jones, Brian Glennon, Philip Donnellan**

Corresponding Author: philip.donnellan@ucd.ie*

School of Chemical & Bioprocess Engineering, University College Dublin, Ireland, D04V1W8

Vapour Concentration Modelling

The vapour of the solvent mixture was known to be a function of the liquid mole fraction of the binary mixture and temperature. Following the method of Renon and Prausnitz [23], the NRTL method was used to predict the activity coefficients for the solvent systems

$$\ln \gamma_1 = {x_2}^2\left[\tau_{21}\left(\frac{G_{21}}{x_1 + x_2 G_{21}}\right)^2 + \frac{\tau_{12} G_{12}}{(x_2 + x_1 G_{12})^2}\right]$$

Equation S1

$$\ln \gamma_2 = {x_1}^2\left[\tau_{12}\left(\frac{G_{12}}{x_2 + x_1 G_{12}}\right)^2 + \frac{\tau_{21} G_{21}}{(x_1 + x_2 G_{21})^2}\right]$$

Equation S2

Where

$$G_{ij} = e^{-\alpha_{ij}\tau_{ij}}$$

Equation S3

$$\tau_{ij} = a_{ij} + \frac{b_{ij}}{T}$$

Equation S4

Where T is temperature in units Kelvin. The value of α_{ij} was 0.3, as was suggested for non-polar substances and polar non-associated liquids.

The values for a_{ij} and b_{ij} were available from Ghmeling, Onken and Arlt [24] and ASPEN [25] and are available in Table S1.

Solvent System	a_{ij}	a_{ji}	b_{ij}	b_{ji}
Acetone/IPA	-2.4106	2.4494	822.489	-583.345
Ethanol/toluene	1.1459	-1.7221	-113.466	992.737

Table S1 – List of NRTL constants for solvent systems studied in this work

The activity coefficients could be calculated across the known binary liquid concentrations and temperatures.

Estimation of reduced vapour pressure by dissolved solute

The vapour pressure modification is described by equation 5.

$$P_{red}{}^{*}{}_{i} = P_i{}^{*} - \frac{\Delta P_i}{10}\left(\frac{Bar}{wt\%\ solid}\right) \times wt\%\ solid \qquad \text{Equation S5}$$

.

It was observed that the vapour pressure reduction (ΔP) was approximately constant within measurable significant figures, across the temperature range at a fixed API concentration

Table S2 - Results from isoteniscope measurements of vapour pressures exerted by 10% w/w solutions of acetone / isopropanol and paracetamol.

Acetone				*Isopropanol*			
Temp (°C)	Reduced P^{vap} (Bar)	Pure P^{vap} (Bar)	ΔP (Bar)	Temp (°C)	Reduced P^{vap} (Bar)	Pure P^{vap} (Bar)	ΔP (Bar)
31	0.360	0.391	0.031	42	0.137	0.155	0.018
33.8	0.400	0.438	0.038	45	0.163	0.182	0.019
37	0.460	0.497	0.037	48	0.195	0.213	0.018
39.5	0.510	0.548	0.035	52	0.235	0.261	0.026
42.5	0.580	0.614	0.034	54	0.261	0.288	0.027
45.2	0.645	0.680	0.033				
50	0.780	0.810	0.030				

It was therefore assumed, within the 0-10% wt/wt solution concentration range and over the measured temperature range, the reduced vapour pressure could be estimated by linear interpolation at predicted concentrations within the measured range (equation 5). It was also assumed that the associated vapour pressure reduction could be applied to each solvent independently, with the reduced pressure value substituting into Raoult's law as:

$$p_i = y_i P \phi_i = \gamma_i x_i P_{red}{}^{*}{}_i$$ *Equation S6*

Gas Chromatography Method

A Shimadzu Nexis FID GC-2030 with a DB-wax column (30 m, 0.25 i.d., d_f = 0.25 µm) was used for the analysis of the acetone/IPA solutions, and a HP-1 column (30 m, 0.32 mm i.d., d_f = 0.1µm) was used for the analysis of the ethanol/toluene solutions. The method was the same for all analyses following the method of Wen and Jetter [21]: the injector port was maintained at 200 °C, the column was maintained at 50 °C for 2 minutes and raised at 50 °C/min to 220 °C and held for a further 2 minutes. The FID detector was maintained at 250 °C and the quantities of the solvents in each sample were measured by integration of the peaks of the responses to obtain the areas. The internal standard method of Grob & Kaiser [22] was applied and 2-butanone was used for all analysis, as it eluted with no overlapping of the analyte peaks. Anisole was the carrier solvent of choice for similar reasoning. Calibration curves were created to obtain the FID relative responses of solvent concentration to a known amount of 2-butanone in the sample. Each sample was prepared using an analytical balance (Mettler Toledo, ME204TE/00); 1 g of the sample solution was weighed into a vial and 0.5g of 2- butanone was added to the same vial. This solution was diluted in 8 mL of Anisole and a small amount of this sample solution (~0.1 mL) was diluted further in a 2 mL vial with more Anisole.

Isoteniscope Measurements

The increasing concentration of dissolved solids in the solution brought about a reduction in the vapour pressure. This would give rise to errors in evaporation rate prediction the higher the concentration of the solute if not accounted for in the model. Following the method of Roche et al [10], the reduction effect of vapour pressure could be easily measured using an isoteniscope device with an associated vacuum pump and thermally controlled water bath. The vapour pressure recorded using the U-tube manometer section of the device was characteristic of the solution's equilibrium vapour pressure at the known temperature and dissolved solute concentration. This was incorporated into the model as a modification to account for the predicted reduction in vapour pressure
